# The Clinical Characteristics and Gene Mutations of Maturity-Onset Diabetes of the Young Type 5 in Sixty-One Patients

**DOI:** 10.3389/fendo.2022.911526

**Published:** 2022-06-30

**Authors:** Shenghui Ge, Mengge Yang, Yuying Cui, Jing Wu, Lusi Xu, Jianjun Dong, Lin Liao

**Affiliations:** ^1^ Department of Endocrinology and Metabology, The First Affiliated Hospital of Shandong First Medical University and Shandong Provincial Qianfoshan Hospital, Jinan, China; ^2^ Cheeloo College of Medicine, Shandong University, Department of Endocrinology and Metabology, Shandong Provincial Qianfoshan Hospital, Shandong Key Laboratory of Rheumatic Disease and Translational medicine, Shandong Institute of Nephrology, Jinan, China; ^3^ College of Traditional Chinese Medicine, Shandong University of Traditional Chinese Medicine, Jinan, China; ^4^ Division of Endocrinology, Department of Internal Medicine, Qilu Hospital of Shandong University, Jinan, China; ^5^ Department of Endocrinology and Metabology, The First Affiliated Hospital of Shandong First Medical University & Shandong Provincial Qianfoshan Hospital, Shandong Key Laboratory of Rheumatic Disease and Translational medicine, Shandong Institute of Nephrology, Jinan, China

**Keywords:** MODY5, diagnosis, HNF1B, gene mutation, renal cysts and diabetes syndrome

## Abstract

**Aims:**

Maturity-onset diabetes of the young type 5 (MODY5), a rare disease, is very easy to be misdiagnosed as type 2 diabetes. To get better understanding of the disease, we analyzed the clinical characteristics and gene mutations of MODY5.

**Methods:**

PubMed, Cochrane, the China National Knowledge Infrastructure, and Wanfang were searched with the following search terms: “MODY5” OR “HNF1B maturity-onset diabetes of the young” OR “maturity-onset diabetes of the young type 5” OR “renal cysts and diabetes syndrome”. Clinical characteristics and gene mutations of MODY5 were analyzed. The demography, clinical characteristics, and blood indicators of patients were described utilizing simple summary statistics. Variables were analyzed by t-test, Wilcoxon signed rank test, and Fisher exact test. Spearman’s correlation analysis was used for bi-variate analysis. All tests were two-sided, and a *p*-value < 0.05 was considered statistically significant. Statistical analysis was performed using the Statistical Package for the Social Sciences version 26 for Windows (SPSS).

**Results:**

A total of 48 literatures were included in this study, including 61 eligible patients and 4 different mutations. Of the 39 patients with available body weight index, 15 (38.46%) were underweight, 21 (53.85%) were normal weight and 3 (7.69%) were overweight or obese. Of the 38 patients with available family history, 25 (65.79%) reported a family history of diabetes. Of the 34 patients with available age of diabetes diagnosis, the median age of diabetes diagnosis was 16.00 years old and 88.24% (30/34) of patients were under 25 years old when they were first diagnosed with diabetes. Renal cysts were presented in 72.41%, hypomagnesemia in 91.67%, and pancreatic dysplasia in 71.88% of the patients. Patients with hepatocyte nuclear factor 1B (HNF1B) deletion had lower serum magnesium, serum creatinine, and higher eGFR than patients with other gene mutations, and the difference was statistically significant.

**Conclusions:**

The young onset of diabetes with low or normal BMI, renal cysts, hypomagnesemia, and pancreatic dysplasia should be recommended to genetic testing in order to differentiate MODY5 from other types of diabetes earlier.

## Introduction

Globally, diabetes is a widely distributed disease which is a chronic metabolic disease that seriously affects human health. Due to advances in genetic testing technologies, an increasing number of monogenic diabetes are differentiated from type 2 or type 1 diabetes. Maturity-onset diabetes of the young (MODY) is a monogenic diabetes accounting for approximately 1% – 2% of diabetes (1), and currently at least 14 types of genes have been confirmed to be associated with MODY, which include *GCK*, *HNF1A*, *HNF1B*, *HNF4A*, *PDX1*, *NEUROD-1*, *KLF-11*, *CEL*, *PAX4*, *INS*, *BLK*, *ABCC8*, *KCNJ11*, *APPL1* (2). The incidence of MODY5 is low, accounting for less than 5% in MODY (3). MODY5 was first described by Horikawa and noted to be related to *HNF1B* (4). Since there was considerable variability in the clinical features of MODY5, it was often misdiagnosed as type 1 diabetes or type 2 diabetes (5). Unfortunately, no systematic summary of MODY5 has been performed to date. In order to get a better understanding of MODY5, this study analyzed the clinical features and gene mutations of MODY5 to help physicians to differentiate MODY5 from other types of diabetes early.

## Materials and Methods

### Data Sources and Study Patients

PubMed, Cochrane, China National Knowledge Infrastructure (CNKI) and Wanfang were searched from the date of inception to February 27, 2022 using the following search terms: “MODY5”, “*HNF1B* maturity-onset diabetes of the young”, “maturity-onset diabetes of the young type 5”, and “renal cysts and diabetes syndrome”. All the enrolled studies met the following criteria: ① the diagnosis of MODY5 was confirmed by genetic test and the mutated sites were described; ② the literature provided the data of FBG or HbA1c; ③ the language of the literature was English or Chinese. The flow chart (**Supplementary Figure 1**) showed the reasons for identification of eligible studies.

The following clinical and laboratory variables were studied: (1) country; (2) gender; (3) age at diagnosis; (4) site and type of gene mutation; (5) family history; (6) BMI; (7) treatment of diabetes; (8) fasting blood-glucose; (9) fasting C-peptide; (10) HbA1c; (11) serum magnesium; (12) other serum indices; (13) renal manifestations; (14) pancreatic manifestations; (15) reproductive manifestations.

### Statistical Analysis

The demography, clinical characteristics, and blood indicators of patients were described utilizing simple summary statistics. Variables were analyzed by t-test, Wilcoxon signed rank test, and Fisher exact test. Spearman’s correlation analysis was used for bi-variate analysis. All tests were two-sided, and a *p*-value < 0.05 was considered statistically significant. Statistical analysis was performed using the Statistical Package for the Social Sciences version 26 for Windows (SPSS).

## Results

### General Data

**Figure 1:**
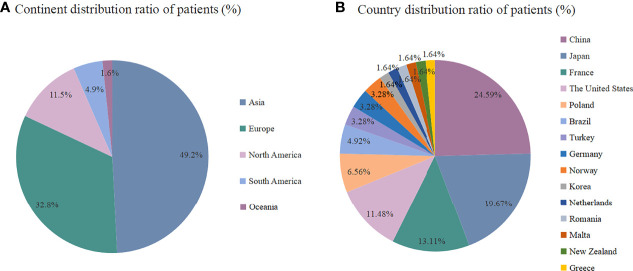
**(A)** Continent distribution ratio among the patients (%), **(B)** Country distribution ratio among the patients (%).

A total of 48 literatures were included in this study, including 61 eligible patients from 15 countries involving 5 continents (**Figure 1**). The top four were China (15/61, 24.59%), Japan (12/61, 19.67%), France (8/61, 13.11%) and The United States (7/61, 11.48%). The patients were distributed in Asia (30/61, 48.2%), Europe (20/61, 32.8%), North America (7/61, 11.5%), South America (3/61, 4.9%) and Oceania (1/61, 1.6%).

### Clinical Features

**Figure 2:**
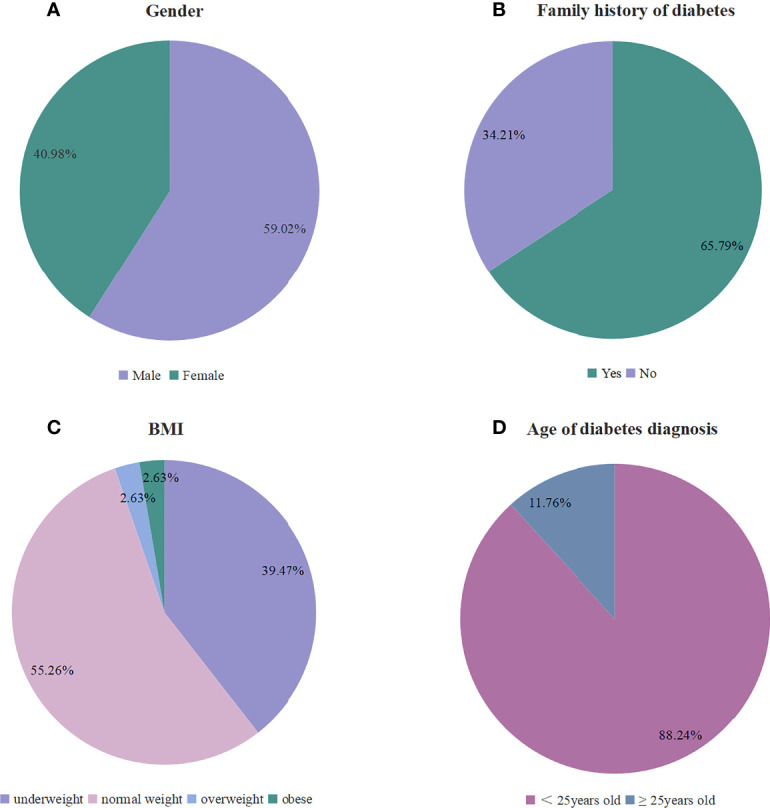
Clinical characteristics of patients with MODY5. **(A–D)** The proportion of several clinical characteristics in enrolled patients: **(A)** gender (N: 61), **(B)** family history of diabetes (N: 38), **(C)** BMI (N: 39), and **(D)** Age of diabetes diagnosis (N: 34).

The clinical data of the patients were shown in **Supplementary Table 1**, **Supplementary Table 2** and **Figure 2**. Among the 61 patients, 36 patients (36/61, 59.02%) were male and 25 patients (25/61, 40.98%) were female. Among the 38 patients mentioning family history, 25 patients (25/38, 65.79%) had a family history of diabetes. A total of 39 patients were recorded with the body-mass-index (BMI). The median of BMI was 19.60 kg/m². According to WHO standard, 15 patients (15/39, 38.46%) were underweight (< 18.5 kg/m^2^), 21 patients (21/39, 53.85%) were normal weight, 2 patients (2/39, 5.13%) were overweight (25~29.9 kg/m^2^), and 1 patient (1/39, 2.56%) was obese (≥ 30 kg/m^2^).

Fasting blood-glucose (FBG) was recorded in 38 patients with a median of 8.37 mmol/L (normal range 3.9-6.1 mmol/L). In addition, 2-hour postprandial blood glucose (2h-PG) was recorded in 9 patients with a mean of 17.24 mmol/L (normal range 4.4-7.8 mmol/L). Fasting C-peptide (FCP) was recorded in 35 patients with a median of 1.23 ng/mL (normal range 1.1-4.4 ng/mL). HbA1c was recorded in 55 patients with a median of 9.40 (normal range 4-6%). Serum magnesium was recorded in 25 patients, with an average of 0.52 mmol/L (normal range 0.70-1.00 mmol/L) and hypomagnesemia occurred in 91.67% of patients. Esti mated glomerularfiltrationrate (eGFR) was recorded in 26 patients with a median of 72.00 ml/min per 1.73m^2^. Serum uric acid level was recorded in 20 patients with an average of 446.01 μmol/L (normal range < 420 μmol/L), of which 13 patients (13/20, 65.00%) had serum uric acid ≥ 420 μmol/L. Serum creatinine was recorded in 34 patients with a median of 96.74 μmol/L (normal range < 106 μmol/L).

Among the 34 patients with recorded age of diabetes diagnosis, the median age of diabetes at diagnosis was 16.00 years old, 88.24% of patients developed diabetes before 25 years old, and 14.71% of patients developed diabetes before 10 years old. There were 10 patients (10/61, 16.39%) had experience of ketoacidosis. Renal morphology was recorded in 58 patients, and 42 patients (42/58, 72.41%) had renal cysts, including left renal cysts (8/42, 19.05%), right renal cysts (3/42, 7.14%), double or multiple renal cysts (25/42, 59.52%), and not classified (6/42, 14.29%). There were 11 patients (11/58, 18.97%) with renal dysplasia, 2 patients (2/58, 3.45%) with renal calculus, 1 patient (1/58, 1.72%) with renal cystic nodules. Pancreatic dysplasia was recorded in 23 patients (23/32, 71.88%), including pancreatic atrophy or agenesis (22/32, 68.75%) and annular pancreas (1/32, 3.13%). Exocrine pancreatic insufficiency was described in 9 patients (9/13, 69.23%), with tests including fecal elastase and p-aminobenzoic acid excretion index. Five patients were with reproductive system abnormalities, including saddle uterus, seminal vesicular cyst with azoospermia, double horn uterus, and double uterus.

The treatments regimens were recorded for 51 patients. Forty-one patients (41/51, 80.39%) were treated with insulin, 6 patients (6/51, 11.76%) underwent diet therapy only and 4 patients (4/51, 7.84%) were given oral hypoglycemic agents without insulin. Among the 41 patients receiving insulin, 4 patients (4/41, 9.76%) combined oral hypoglycemic drugs, 1 patient (1/41, 2.44%) combined liraglutide and 36 patients (36/41, 88.80%) received insulin monotherapy.

The clinical data of patients with *HNF1B* deletion and other mutations were shown in **Supplementary Table 3**. In patients with *HNF1B* deletion, the median of FBG was 8.67 mmol/L, FCP was 1.40 ng/mL, serum creatinine was 84.86 μmol/L, and the mean of serum magnesium was 0.48 mmol/L, serum uric acid was 429.15 μmol/L. In patients with other mutations, the median of FBG was 7.82 mmol/L, FCP was 1.08 ng/mL, serum creatinine was 134.37 μmol/L, and the mean of serum magnesium was 0.61 mmol/L, serum uric acid was 466.62 μmol/L. Patients with *HNF1B* deletion had lower serum magnesium, serum creatinine, and higher eGFR than patients with other gene mutations, and the difference was statistically significant. However, there was no significant difference in the incidence of pancreatic dysplasia.

**Figure 3:**
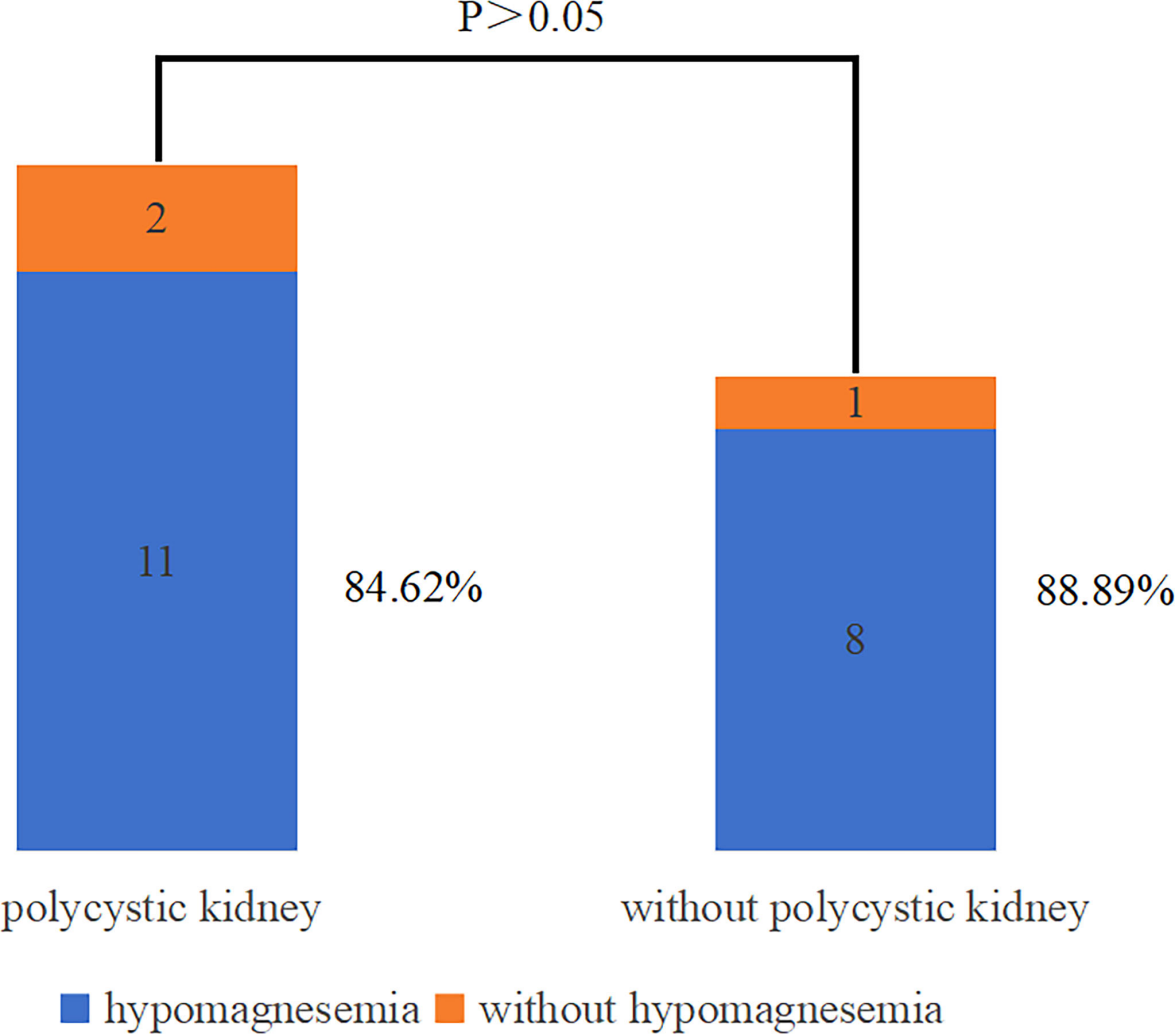
Correlation analysis of hypomagnesemia and polycystic kidney disease in MODY5 patients.

The correlation analysis of renal cysts and serum parameters was shown in **Table 1**. In MODY5, there was no correlation between the occurrence of renal cysts and eGFR, serum creatinine, serum magnesium, and serum uric acid. The proportion of hypomagnesemia in MODY5 patients with or without polycystic kidney disease was presented in **Figure 3**. Hypomagnesemia was presented in 11 patients (11/13, 84.62%) with polycystic kidney disease. And hypomagnesemia was presented in 8 patients (8/9, 88.89%) without polycystic kidney disease. There was no significant correlation between polycystic kidney disease and hypomagnesemia in MODY5.

**Table 1:** Spearman’s correlation analysis evaluating the association between serum parameters and renal cysts of MODY5 patients.

Subjects	Case	Renal cysts (yes/no)	
		r	P
eGFR	25	0.198	0.344
Serum creatinine	32	-0.087	0.638
Serum magnesium	23	0.329	0.125
Serum uric acid	20	-0.310	0.183

eGFR, estimated glomerularfiltrationrate.

### Gene Mutations

**Figure 4:**
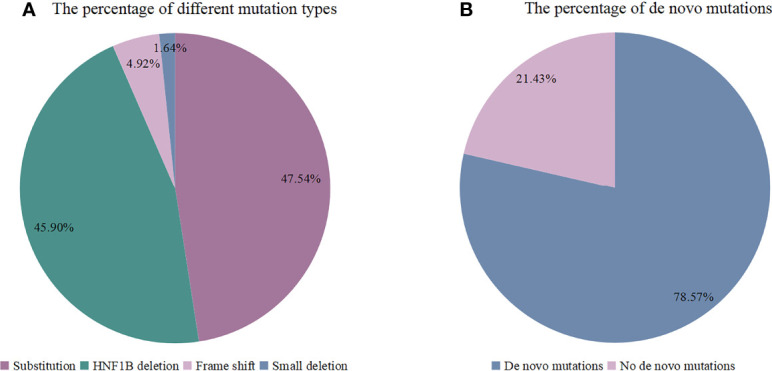
**(A)** The percentage of different mutation types (N: 61), **(B)** The percentage of *de novo* mutations (N: 14).

The gene mutations of the patients were shown in **Table 2** and **Figure 4**. Totally 4 types of mutations were identified, which included substitution (29/61, 47.54%), *HNF1B* deletion (28/61, 45.90%), frame shift (3/61, 4.92%) and small deletion (1/61, 1.64%). The genetic information of the patient’s parents was recorded in 14 cases, and *de novo* mutations were confirmed in 11 patients (11/14, 78.57%).

**Table 2:** *HNF1B* mutations of MODY5 patients.

No.	Country	Gender	cDNA	Protein
1	China	F	c.1007A>G	p.His336Arg
2	The United States	F	c.1127C>T	p.Thr376Ile
3	China	M	c.1132C>T	p.Gln378*
4	Japan	M	c.457C>A	p.His153Asn
5	Malta	F	c.1580G>A	p.Arg527Gln
6	Japan	M	c.434T>A	p.Leu145Gln
7	France	F	c.443C>G	p.Ser148Trp
8	China	M	c.443C>T	p.Ser148Leu
9	The United States	M	c.443C>T	p.Ser148Leu
10	Turkey	F	c.443C>T	p.Ser148Leu
11	Germany	M	c.443C>T	p.Ser148Leu
12	Korea	F	c.476C>T	p.Pro159Leu
13	China	F	c.494G>A	p.Arg165His
14	Japan	M	c.503T>C	p.Leu168Pro
15	China	M	c.530G>A	p.Arg177Gln
16	China	F	c.541C>T	p.Arg181*
17	Rumania	M	c.715G>C	p.Gly239Arg
18	Poland	M	c.742C>T	p.Gln248*
19	Poland	M	c.742C>T	p.Gln248*
20	China	F	c.826C>T	p.Arg276*
21	Japan	M	c.826C>T	p.Arg276*
22	France	M	c.884G>A	p.Arg295His
23	France	M	c.884G>A	p.Arg295His
24	France	M	c.143delT	p.Leu48fs
25	Brazil	M	c.983delC	p.Pro328Leufs*48
26	Norway	F	c.409_483del	p.Arg137_Lys161del
27	The United States	F	c.1149delinsTGGCC	p.Arg351Leufs*10
28	Japan	F		p.Leu264Ser
29	Norway	M		p.Phe148Leu
30	Poland	M	c.1046-15T>A	
31	France	M	c.544+4A>C	
32	Japan	F	c.544+1G>T	
33	Japan	M	g.37731831C>G	
34	China	F	*HNF1B* deletion	
35	China	M	*HNF1B* deletion	
36	Japan	F	*HNF1B* deletion	
37	Japan	M	*HNF1B* deletion	
38	The United States	F	*HNF1B* deletion	
39	France	M	*HNF1B* deletion	
40	France	F	*HNF1B* deletion	
41	Brazil	M	*HNF1B* deletion	
42	Brazil	M	*HNF1B* deletion	
43	New Zealand	F	*HNF1B* deletion	
44	China	M	*HNF1B* deletion ◇	
45	China	F	*HNF1B* deletion ◇	
46	China	F	*HNF1B* deletion ◇	
47	China	M	*HNF1B* deletion ◇	
48	China	F	*HNF1B* deletion ◇	
49	Japan	F	*HNF1B* deletion ◇	
50	Japan	F	*HNF1B* deletion ◇	
51	Japan	M	*HNF1B* deletion ◇	
52	The United States	M	*HNF1B* deletion ◇	
53	The United States	M	*HNF1B* deletion ◇	
54	The United States	M	*HNF1B* deletion ◇	
55	France	M	*HNF1B* deletion ◇	
56	Poland	M	*HNF1B* deletion ◇	
57	Turkey	M	*HNF1B* deletion ◇	
58	Germany	F	*HNF1B* deletion ◇	
59	Netherlands	M	*HNF1B* deletion ◇	
60	China	M	*HNF1B* deletion §	
61	Greece	F	*HNF1B* deletion ¶	

Gender: F, female; M, male. § Exon2~9del. ◇HNF1B deletion is accompanied with 17q12 deletion. ¶HNF1B deletion is accompanied with a HNF1A mutation (c.685C>T, p.Arg229*).

## Discussion

MODY5 was caused by *HNF1B* mutations due to changes in its effectors. Since MODY5 was first described, there have been some reports about this disease. However, most reports were case reports. Unlike previous studies, our study summarized the clinical features and genetic mutations of 61 patients with MODY5, and demonstrated the MODY5 patients had the following clinical characteristics: (1) high incidence of renal cysts (72.41%), hypomagnesemia (91.67%), and pancreatic dysplasia (71.88%); (2) early onset of diabetes; (3) normal or underweight (92.31%).

As for diagnosis, most MODY5 patients were misdiagnosed as type 1 diabetes or type 2 diabetes (5). Therefore, it was crucial to identify the clinical features of diabetes in MODY5 patients. Our study showed that most patients (88.24%) developed diabetes before 25 years old, and the median onset age of diabetes in MODY5 patients were 16.00 years old, which was different from previous studies (6–9). A multi-clinical center study showed that the onset age of diabetes in MODY5 patients were more than 25 years old (6). Another multicenter retrospective cohort study of patients with *HNF1B* mutation showed that the mean age of diagnosis of diabetes was 26 years old (7). In the Japanese study (8), only 6% of patients developed diabetes during adolescence. Lim et al. reported 4 patients (29%) developed diabetes before 14.6 years old (9). The reasons for these different conclusions might be as follows: The results might be affected by different inclusion criteria. In our study, glucose-related data must be recorded when data literatures were included. While in the most above-mentioned study, age was set in the inclusion criteria. Notably, although the onset of diabetes in most patients was under 25 years old, only 14.71% of patients developed diabetes before 10 years old. Teo et al. indicated that compensatory mechanisms in the pancreatic transcription factor network due to *HNF1B* mutations (10). Therefore, MODY5 often occurred in adolescence rather than in the neonatal period. Different mechanisms were involved in the development of MODY5 diabetes. Firstly, pancreatic dysplasia was presented in most patients (71.88%). A study in monozygotic twins with MODY5 showed that impaired insulin secretion was not due to pancreatic B cell functional defects, but pancreatic dysplasia (11). It has also been previously reported that pancreatic dorsal dysplasia was associated with *HNF1B* mutations (12). Therefore, pancreatic dysplasia might be involved in the development of diabetes. Secondly, some mutations might be related to changes in GLUT2-related signaling pathways, such as p.Arg276* mutation and p.Pro159Leu mutation (13). Previous studies have shown that glucose-stimulated insulin secretion was significantly reduced when the p.Arg276* mutation occurred (14). However, this change in insulin secretion was not presented in KCl stimulation. Because *HNF1B* might play a role in intracellular ATP production and indirectly regulate K^+^ current through ATP-sensitive K^+^ channels (14). There were individual differences in diabetes among MODY5 patients. This also explained why MODY5 was easily misdiagnosed as type 1 diabetes or type 2 diabetes.

The renal manifestations were prominent in MODY5 patients. Renal cysts (72.41%) were the most common morphological abnormalities, with the majority presented as multiple renal cysts. Although multiple renal cysts were a common renal phenotype in MODY5 patients, they needed to be differentiated from other polycystic kidney diseases. In a study of children with cystic kidney disease (15), hypomagnesemia appeared to be a marker of differential diagnosis between autosomal dominant polycystic kidney disease (ADPKD), autosomal recessive polycystic kidney disease (ARPKD) and MODY5. Patients with MODY5 often had hypomagnesemia, but it was uncommon in patients with ADPKD or ARPKD. Hypomagnesemia was presented in 84.62% of MODY5 patients with multiple renal cysts. Therefore, multiple renal cysts with hypomagnesemia should raise suspicion for MODY5 diagnosis. In addition, the correlation between hypomagnesemia and multiple renal cysts was analyzed in MODY5 patients. The results showed that there was no significant relationship between hypomagnesemia and multiple renal cysts. At the same time, the correlation between serum magnesium and renal cysts was studied in MODY5 patients, and no association was presented. Therefore, hypomagnesemia might be helpful to differentiate different polycystic kidney diseases. This depended on the high incidence of hypomagnesemia in MODY5 rather than polycystic kidney itself.

The renal function of MODY5 varied greatly from normal to end-stage renal failure (16), but there was no unified conclusion about the correlation between gene mutation types and renal function. In our study, patients with a total gene deletion had a better renal prognosis than those with other gene mutations. Firstly, additional deleted genes within *HNF1B* might include genes that impair renal functional. But the effect has not been reported thus far (6). Secondly, patients with *HNF1B* deletion may be affected by 17q12 deletion. This led to underexpression of certain genes and indirectly reduced kidney damage (17). But another study showed no correlation between genotype and renal functional (9). Although renal malformations appeared to be the common manifestation of *HNF1B* mutations, progression to end-stage renal disease (ESKD) in patients with *HNF1B* mutations seemed to be rare (18). The specific mechanism needs to be further explored. Furthermore, our study did not find an association between renal function and renal cysts. Renal function might not be affected by renal cysts.

Pancreatic abnormalities in MODY5 were mainly dysplasia. Ventral pancreatic dysplasia was the most prominent, because *HNF1B* was related to ventral pancreatic development, its mutation could cause pancreatic dysplasia (19–21). It was also reported that patients might have pancreas divisum, intraductal papillary mucinous tumo, yet these manifestations were extremely rare (6). In MODY5, in addition to insufficient insulin secretion due to pancreatic dysplasia, pancreatic exocrine function was also often impaired. Pancreatic exocrine dysfunction was also presented in MODY5 patients. These patients might present with abdominal pain, loose stools and weight loss. Fecal elastase was a convenient indicator of pancreatic exocrine function (22). Therefore, fecal elastase test was recommended for patients with suspected MODY5.

Hypomagnesemia was common in patients with MODY5. The deficient *HNF1B* downregulated the expression of *FXYD2.* The downregulated *FXYD2* blocked the encoding of the γ subunit of the Na^+^-K^+^-ATPase and indirectly led to hypomagnesemia (23–25). Although hypomagnesemia was a common presentation in patients with MODY5, patients had varying degrees of hypomagnesemia in different mutation types. Patients with *HNF1B* deletion had worse hypomagnesemia than those with other mutations. The reason might be that patients with other mutations have worse renal function relative to patients with HNF1B deletion, which might reduce renal magnesium loss (15). Therefore, this should be fully considered in patients with renal insufficiency.

In addition, patients with MODY5 were prone to hyperuricemia, and some patients developed gout. Hyperuricemia is defined as serum uric acid level ≥ 420 μmol/L. In our study, 65.00% of MODY5 patients met the diagnosis of hyperuricemia. Previous studies indicated that hyperuricemia was a common early manifestation of MODY5 in children, but its utility as a predictor of the disease was limited (26). However, hyperuricemia might serve as a supplement to raise our suspicions about MODY5.

In our study, reproductive system abnormalities presented in 5 patients, included saddle uterus, eminal vesicular cyst with azoospermia, double horn uterus, and double uterus. Evaluation of the reproductive system was necessary in patients with reproductive needs or the young people. The correlation between neurological abnormalities and *HNF1B* was still unclear. Currently, it was generally believed that neurological abnormalities were more likely to come from genes other than *HNF1B* (27). Therefore, it was necessary to consider expanding the scope of genetic testing when neurological symptoms were prominent.

Clinical management is critical for MODY5 patients. Our study showed that most of the MODY5 patients (80.39%) received insulin therapy. Only a minority of patients did not use insulin after the onset of diabetes. Sulfonylureas were suitable for patients with a certain reserve of pancreas islet function (28), and they were not suitable for patients with severe pancreatic dysplasia. Notably, recent reports have provided some new therapeutic possibilities. A patient with *HNF1B* deficiency MODY5 who was treated with liraglutide restored endogenous insulin secretion and stopped insulin injection (29). This might be due to the upregulation of *PAX6* by glucagon-like peptide 1 receptor agonists (30), which promoted the regeneration of insulin-secreting cells. In addition, MODY5 patients might benefit from Bacillus Calmette – Guerin (BCG) vaccination (31). Studies showed that BCG treatment might regenerate pancreatic B cell (32, 33). This might compensate for the deficiency caused by pancreatic dysplasia. Management of pancreatic exocrine dysfunction is often neglected. Early pancreatic replacement therapy for these patients can improve their symptoms and normalize their weight (22). For hypomagnesemia patients, inorganic magnesium treatment was less effective, there were side effects of diarrhea. Organomagnesium such as magnesium aspartate was recommended (34). At the same time, thiazide diuretics should be carefully used in patients with hypomagnesemia, which could aggravate hypomagnesemia (35). In MODY5 patients with hyperuricemia, drugs that promote uric acid excretion were prohibited. Allopurinols were recommended to control serum uric acid levels and delay renal damage (36).

Our study has several limitations. Firstly, in order to comprehensively understand the clinical characteristics of MODY5 patients, all articles were limited to at least recording diabetes-related indicators, which might lead to selection bias in our study. Secondly, because of the low incidence of MODY5, some rare clinical manifestations are difficult to analyze. Finally, the mechanism of different mutations leading to various clinical features still remains confused and further studies are needed to explain its molecular mechanism.

In summary, our study shows that MODY5 often has multiple clinical manifestations. Diabetes usually starts before 25 years old and often with pancreatic dysplasia. Patients with *HNF1B* deletion have a better renal prognosis and worse hypomagnesemia than patients with other gene mutations. The young onset of diabetes with low or normal BMI, renal cysts, hypomagnesemia, and pancreatic dysplasia should be recommended to genetic testing earlier in order to differentiate MODY5 from other types of diabetes.

## Data Availability Statement

The original contributions presented in the study are included in the article/**Supplementary Material**. Further inquiries can be directed to the corresponding author.

## Author Contributions

SG: Document Retrieval, Data Extraction, Data analysis, Essay writing, and Paper submission. MY and YC: Data analysis. JW and LX: Data Extraction. JD and LL: Article innovation and Paper submission. All authors contributed to the article and approved the submitted version.

## Funding

This work was funded by the National Natural Science Foundation of China (82170847).

## Conflict of Interest

We declare that the research was conducted in the absence of any commercial or financial relationships that could be construed as a potential conflict of interest.

## Publisher’s Note

All claims expressed in this article are solely those of the authors and do not necessarily represent those of their affiliated organizations, or those of the publisher, the editors and the reviewers. Any product that may be evaluated in this article, or claim that may be made by its manufacturer, is not guaranteed or endorsed by the publisher.
