# Supplementary material for: The Clinical Characteristics and Gene Mutations of Maturity-Onset Diabetes of the Young Type 5 in Sixty-One Patients
Source: Front Endocrinol (Lausanne). 2022 Jun 30;13:911526. doi: 10.3389/fendo.2022.911526 (PMC9281895; doi:10.3389/fendo.2022.911526)
Supplement: Supplementary file 1 [file DataSheet_1.doc]

Figure S1 Literature review inclusion process.

Records identified through database searches (n=555)

Records after duplicates removed (n=464)

355 articles excluded based on screening of titles and abstracts for inclusion

Articles included in the final analysis (n=48)

Full-text articles obtained for further review (n=109)

40 articles excluded without complete information of patients

15 excluded articles: non-case report

6 excluded articles: no diabetes

**TABLE S1 |** Summary of the clinical features of MODY5 patients.

| Subjects | No. of patients | Data |
| --- | --- | --- |
| Onset (Year) | 34 | 16.00 (11.75-23.00) |
| BMI (Kg/m²) | 39 | 19.60 (17.30-22.40) |
| FBG (mmol/L) | 38 | 8.37 (6.37-13.69) |
| FCP (ng/ml) | 35 | 1.23 (0.68-2.22) |
| 2h-BG (mmol/L) | 9 | 17.24 ± 6.06 |
| HbA1c (%) | 55 | 9.40 (6.60-13.20) |
| Serum magnesium (mmol/L) | 25 | 0.52 ± 0.12 |
| Serum sodium (mmol/L) | 9 | 140.44 ± 3.40 |
| Serum [potassium](javascript:;) (mmol/L) | 11 | 3.74 ± 0.42 |
| Serum calcium (mmol/L) | 11 | 2.34 ± 0.21 |
| Serum creatinine (μmol/L) | 34 | 96.74 (74.15-149.81) |
| Serum uric acid (μmol/L) | 20 | 446.01 ± 88.12 |
| eGFR (ml/min per 1.73m^2^) | 26 | 72.00 (56.70-93.48) |

Results are expressed as mean ± standard deviation, or as median (IQR).

BMI = body mass index, FBG = fasting blood glucose, FCP = fasting c-peptide, 2h-BG = 2-hour postprandial blood glucose, HbA1c = glycosylated hemoglobin, eGFR = esti mated glomerularfiltrationrate.

**TABLE S2 |** Clinical phenotypes in MODY5 patients.

| **Clinical phenotype** | **Features: numbers of cases** | **Observed prevalence,**  **n/N investigated (%)** |
| --- | --- | --- |
| **Kidney** |  |  |
| Renal cysts | Left renal cysts: 8  Right renal cysts: 3  Double or multiple renal cysts: 25  Not classified: 6 | 42/58 (72.41) |
| Renal dysplasia |  | 11/58 (18.97) |
| Renal calculus |  | 2/58 (3.45) |
| Renal cystic nodules |  | 1/58 (1.72) |
| **Pancreas** |  |  |
| Pancreatic atrophy or agenesis |  | 22/32 (68.75) |
| Annular pancreas |  | 1/32 (3.13) |
| Pancreatic exocrine dysfunction |  | 9/13 (69.23) |
| **Hypomagnesemia** |  | 22/24 (91.67) |
| **Hyperuricemia** |  | 13/20 (65.00) |
| [**Genital**](javascript:;) [**system**](javascript:;) | Saddle uterus: 1  Seminal vesicular cyst with azoospermia: 1  Double horn uterus: 2 Double uterus: 1 | 5/8 (63.64) |
| **ketoacidosis** |  | 10/61 (16.39) |

Results are expressed as actual numbers and percentages.

| **TABLE S3 |** Comparison of clinical features between different genotypes. | | | | | | |
| --- | --- | --- | --- | --- | --- | --- |
| Subjects | Deletion | |  | Mutations | | P |
|  | No. of patients | Data |  | No. of patients | Data |  |
| Onset (Year) | 13 | 20.00 (14.50-23.50) |  | 21 | 14.00 (10.50-22.50) | 0.114 |
| BMI (Kg/m²) | 16 | 18.57 (17.29-20.00) |  | 23 | 20.50 (17.60-23.40) | 0.072 |
| FBG (mmol/L) | 18 | 8.67 (7.30-15.74) |  | 20 | 7.82 (5.57-10.91) | 0.174 |
| 2h-BG (mmol/L) | 4 | 20.47 ± 5.39 |  | 5 | 14.66 ± 5.73 | 0.221 |
| FCP (ng/ml) | 15 | 1.40 (0.30-2.80) |  | 20 | 1.08 (0.74-1.43) | 0.571 |
| HbA1c (％) | 26 | 11.20 (6.68-16.18) |  | 29 | 8.40 (6.20-11.80) | 0.077 |
| Serum magnesium (mmol/L) | 17 | 0.48 ± 0.10 |  | 8 | 0.61 ± 0.13 | 0.008 ** |
| Serum sodium (mmol/L) | 7 | 140.14 ± 3.85 |  | 2 | 141.50 ± 0.71 | 0.650 |
| Serum [potassium](javascript:;) (mmol/L) | 8 | 3.73 ± 0.35 |  | 3 | 3.77 ± 0.67 | 0.892 |
| Serum calcium (mmol/L) | 10 | 2.33 ± 0.22 |  | 1 | 2.5 | 0.474 |
| Serum creatinine (μmol/L) | 15 | 84.86 (61.10-103.43) |  | 19 | 134.37 (91.00-182.00) | 0.019 * |
| eGFR (ml/min per 1.73m²) | 13 | 81.00 (68.15-114.30) |  | 13 | 58.00 (23.00-83.00) | 0.043 * |
| Serum uric acid (μmol/L) | 11 | 429.15 ± 103.62 |  | 9 | 466.62 ± 64.42 | 0.358 |
| Pancreatic morphology | 17 | Normal = 4  [Abnormal](javascript:;) = 13 |  | 15 | Normal = 6  [Abnormal](javascript:;) = 9 | ＞0.05 |
| Results are expressed as mean ± standard deviation, or as median (IQR).  BMI = body mass index, FBG = fasting blood glucose, FCP = fasting c-peptide, 2h-BG = 2-hour postprandial blood glucose, HbA1c = glycosylated hemoglobin, eGFR = esti mated glomerularfiltrationrate.  * *p* < 0.05, ** *p* < 0.01. | | | | | | |
